# Marketplace and Literature Review of Spanish Language Mental Health Apps

**DOI:** 10.3389/fdgth.2021.615366

**Published:** 2021-02-15

**Authors:** Alma Oñate Muñoz, Erica Camacho, John Torous

**Affiliations:** ^1^Harvard Medical School, Boston, MA, United States; ^2^Beth Israel Deaconess Medical Center, Boston, MA, United States

**Keywords:** mHealth, culture, Spanish, apps, technology

## Abstract

Language differences between patients and providers remains a barrier to accessing health care, especially mental health services. One potential solution to reduce inequities for patients that speak different languages and improve their access to care is through the delivery of healthcare through mobile technology. Given that the Latinx community serves as the largest ethnic minority in the United States, this two-phased review examines Spanish app development, feasibility and efficacy. Phase 1 explored the commercial marketplace for apps available in Spanish, while phase 2 involved a literature review of published research centered around the creation, functions, and usability of these apps using the PubMed and Google Scholar electronic databases. Of the apps available on the database, only 14.5% of them had Spanish operability. The literature search uncovered 629 results, of which 12 research articles that tested or described 10 apps met the inclusion criteria. Of the 10 apps studied in this literature review, only four apps were translated to Spanish. Our study reveals that despite increasing interest in Spanish-language apps to address mental health, the commercial marketplace is not currently meeting the demand.

## Introduction

Latinos are the largest ethnic minority in the United States and currently account for 18.5% of the US population (1). It is estimated that 21.3% or nearly 13 million Latinx individuals have at least one mental illness (1, 2). Yet, despite the great need for assessment and treatment of mental health illness in the Latinx population, data shows that only 9.6% of them accessed any mental health services (2). There is a growing body of literature that highlights the barriers to accessing mental health care, which includes low rates of insurance coverage, legal status, stigma, and socioeconomic factors. Language is a salient factor in accessing mental health care services. A study in 2013 showed that 32% or 15.7 million Latinx individuals report speaking English less than “very well.” (3) Language differences between patients and providers have been shown to be detrimental for effective communication, which can lead to lower quality of care, and poorer outcomes (4, 5). The imbalance between demand and supply is aggravated by the fact that only an estimated 4.0% of psychiatrists are Latinx (6).

One potential solution to reduce inequities for Spanish-speaking patients and improve access to care is through the delivery of healthcare through mobile health care (*m*Health). Through the use of apps via mobile devices, patients can access a wealth of resources to improve their mental health. This change requires both access to a smartphone with internet capabilities and a desire of Latinx to use *m*Health; both are true today. Research shows that 80% of Latinx individuals have access to the internet via a mobile device, thus making delivery of *m*Health feasible (7, 8). Latinx individuals want *m*Health as demonstrated in a 2016 study where over 85% of participants reported interest in using mobile apps to improve their health and at least a quarter stated they would use a mobile app for mental wellness (9).

Despite the demand for more mHealth for Spanish-speaking populations, it is unclear whether the commercial marketplace has met the increasing need and the extent of published literature on Spanish app development, feasibility and efficacy are unknown. The objectives of this U.S. app store marketplace and literature review are to in phase (1) review the number and characteristics of Spanish mental health apps available on the commercial market; in phase (2) review existing literature on the development, translation, and cultural adaptation of mental health apps to Spanish-language/culture as well as the feasibility and efficacy of these apps in the Spanish-speaking population; (3) synthesize the results from the first two phases to identify key challenges, opportunities, and recommendations for development, translation, and cultural adaptation of Spanish-language mental health apps.

## Methods

### Phase 1

This review was conducted in two phases. Phase 1 involved searching a database of commercial market for apps available in Spanish, while phase 2 consisted of a literature review of published research centered around the creation, functions, and usability of these apps. To determine the commercial market's availability of mental health apps in Spanish, we utilized the Mobile Health Index and Navigation (MIND) database published by The Division of Digital Psychiatry at BIDMC (available at apps.digitalpsych.org). The database currently reports the existence of 220 mental health apps in the commercial market and collects data on 105 objective questions set forth by the APA for each registered mental health app. It is a useful resource for this phase as it is the largest database of mental health apps supported by peer reviewed evidence, publicly accessible, and allows users to filter through apps based on personal preferences and priorities (10). We utilized this database and filtered results by “Spanish Functionality” to find all the apps that are currently available in Spanish and collected basic characteristics of these apps, including platform availabilities, supported conditions, engagement features, connection to other services, and supporting study availability, see Table 1.

**Table 1:** Characteristics of mental health mobile apps in Spanish.

	**Characteristic**	**n**	**%**
	Number of Apps	32	100%
Platforms	iOS	31	96.9%
	Android	27	84.4%
	Companion website for App	8	25.0%
Supported conditions	Stress & anxiety	16	50.0%
	Mood disorders	14	43.8%
	Sleep	9	28.1%
	Phobias	6	18.8%
	Eating disorders	6	18.8%
	OCD	5	15.6%
	Personality disorders	5	15.6%
	Schizophrenia	4	12.5%
Engagement	Audio/music/scripts	16	50.0%
	Gamification	14	43.8%
	Videos	9	28.1%
Connection to other services	Link to formal care/coaching	10	31.3%
	Crisis management feature	8	25.0%

### Phase 2

To understand the current state of mental health apps in Spanish, we created a searchable question to identify Spanish-language app development, feasibility, and efficacy. The developed search strategy was conducted on two electronic databases: PubMed and Google Scholar. Major themes searched included Spanish, Spanish-language, Spanish-speaking, Hispanic, and Latinx combined with health and mental health synonyms. This was joined to a list of applicable terms for the type of technology utilized, including mobile devices, smartphones, and apps. Given the fast-paced changes in technology, only articles published after January 2015 were included. To be included, papers had to meet the following criteria: (1) feature a Spanish-language mobile app, (2) describe the development, feasibility, or interventional approach of a mobile app, and (3) the app must address at least one mental health disorder including, but not limited to depression, anxiety, substance use disorder, and/or eating disorders. Articles were excluded if (1) published before January 2015, and (2) app does not address any mental health disorders.

The search revealed 629 articles. A review of the 629 citations/abstracts was conducted manually. The full text was considered for papers with abstracts unavailable. This initial screening resulted in 49 articles that met the preliminary inclusion criteria. A snowball approach was used to ensure the literature search was comprehensive. This involved reviewing papers that cited the 49 articles, searching for previous papers written by the lead authors, and utilizing the “related articles” feature on each database. Following a full-text review of the final set of papers, 12 met the comprehensive criteria and are reviewed here. There are no conflicts of interest identified by the authors of this study. All authors certify responsibility for the manuscript.

## Results

### Phase 1

A review of the Division of Digital Psychiatry's mHealtgh App Navigation Database (MIND) revealed that of 220 mental health apps available on the commercial market, 32 (14.5%) offer Spanish operability (10). Of these, 31 (96.9%) are available on iOS and 27 (84.4%) are available on Android. The most common supported conditions were stress and anxiety (*n* = 16, 50.0%) and mood disorders (*n* = 14, 43.8%). Significant overlap was noted with 12 (37.5%) apps supporting both mood and stress and anxiety conditions. Of the 32 apps, 10 (31.3%) have supporting studies published (11–32). However, a review of these studies revealed that none of them focused on Spanish-speaking populations. Further, none of the apps on the marketplace found in Phase 1 were found in the app mentioned in the literature review of Phase 2 as outlined below. Table 1 summarizes the most salient characteristics of these apps.

### Phase 2

The literature search revealed 12 research articles that tested or described 10 apps. Among the 10 apps, only four distinct mental health disorders were addressed. Of the 12 articles, six described app development and/or cultural adaptation, three studied feasibility, and three described interventional studies. Most articles (*n* = 8, 66.7%) focused on Spanish-speaking Latino adults in the United States. The rest of the articles focused on Spanish-speaking individuals in Peru, Colombia, and Australia. Of the 10 apps described or studied, half (*n* = 5, 50.0%) aimed to address depressive symptoms. Table 2 displays the article and app characteristics including articles' research design and study population, as well as the apps' supported conditions. Of note, control apps not studied or reported on were not analyzed in Table 2.

**Table 2:** Summary metrics of studies on mental health apps in Spanish.

		**Studies**	**%**
**Journal article characteristics (*****n*** **= 12)**			
Research design	App development protocol	6 (33–38)	50.0%
	Feasibility	3 (39–41)	25.0%
	Interventional	3 (42–44)	25.0%
Study Population	US Spanish-Speaking Latinos	8 (33, 36–38, 40, 42–44)	66.7%
	Peru	2 (39, 41)	16.7%
	Colombia	1 (34)	8.3%
	Australian Spanish-Speaking Latinos	1 (35)	8.3%
**App characteristics (*****n*** **= 10)**			
Mental health disorder addressed by the App	Depression	5 (33, 39–42, 44)	50.0%
	Substance use disorder	3 (36, 38, 43)	30.0%
	General mental health & emergency services	1 (34, 35)	10.0%
	Eating disorders	1 (37)	10.0%

#### App Development Protocol

The ¡Aptívate! app utilizes Brief Behavioral Activation (BBA) to address depressive symptoms by emphasizing that behaviors can influence mood and encourages participants to complete activities that align with self-selected values. To create this app, researchers started with the English app “Moodivate” and translated the app interface into Spanish utilizing a back-translation approach with the help of bilingual translators. Given that the app has patients self-select values and activities, cultural adaptation was not deemed necessary. However, the psychoeducation component of the app was carefully designed to avoid stigma in this population by de-emphasizing depressive symptoms as internal flaws and emphasizing the lack of environmental rewards as the etiology (33).

The Mental Health eClinic (MHeC) app was developed to address a broad range of mental health concerns and includes a triage system for those needing urgent help. The app was developed utilizing a participatory design methodology for Spanish-speaking youth in two different settings: international students in Australia and native youth in Colombia (34, 35). In a participatory design, stakeholders including patients, supportive others, and healthcare professionals provide their input in all six phases of app development. In both settings, all developmental phases that directly involved stakeholders were conducted in Spanish, eliminating the need for translation. In Australia, cultural adaptation included changing of the language question to specify Spanish dialects, changing the ethnicity question to reflect indigenous populations, and after initial disagreement, adoption the informal “tú” throughout the app (35). In Colombia, cultural adaptation included incorporating family structure and support networks, establishing credibility through university, health service provider, and community organization collaboration, and given the country's characteristics screening for economic stability, food security, and violence exposure (34).

The Automated Bilingual Computerized Alcohol Screening and Intervention (AB-CASI) mobile app was developed as the Spanish version of the emergency department-based alcohol screening, brief intervention, and referral to treatment (ED-SBIRT) program (36). The goal of the app is to address alcohol use disorders in Spanish without requiring extensive human resources, such as translators, in the emergency department. To culturally adapt this app, the researchers utilized user-centered design through design, development, and evaluation of app prototypes. This methodology ensures that stakeholders, namely patients and professionals, are involved in the development process. This app also addresses literacy issues through text-to-speech, which was found to be crucial for culturally adapting the app. Text to speech apps transform written text into audio. Researchers ultimately chose a Text-To-Speech app to help with this process. Additionally, this work emphasizes that beyond translation and cultural adaptation, it is necessary to adapt health apps to the context in which they will be used. In this instance, the app had to be designed for use in an emergency department, therefore the app must have capability to save progress and start/stop/pause.

The Ecological Momentary Assessment (EMA) app was created to understand unhealthy eating and weight control behaviors of Mexican American women with low literacy (37). The app aims to collect information on these activities repeatedly in its natural context without having to rely on memory. Original studies of this app were geared toward college-enrolled women, but in contrast this study focused on women with low health literacy, requiring a shift from written components to pictures, icons, and sound features. App development was achieved by utilizing a user-centered methodology which involved the end-users at all four stages of development. Through this work, researchers found that: (1) text-to-icon translation (words are translated into images) was more complex than anticipated given the discrepancy in definitions of unhealthy eating behaviors between participants and researchers; (2) participants described forms of weight control products as opposed to their intended effects (ex: diet pill as opposed to laxative); (3) icons were found to be too complex to use to collect context and mood, therefore this component was not included in the final app.

Finally, Muñoz et al. submitted a study protocol for the design, development, and evaluation of the San Francisco Stop Smoking app which aims to help Latinos stop smoking (38). Although results of this study are pending, the protocol describes the human-centered methodology utilized to create this app. To achieve cultural adaptation, the first phase of development will require field observations of potential app users. These observations will give researchers first-hand information about how Latinos use their phones, how they interact with apps, and ultimately help researchers understand what app features might best serve the end-users. Researchers will also conduct workshops with Spanish speaking patient in which they will design an ideal app. This will also give researchers direct feedback on specific features that might be needed for the app to work for Spanish-speakers trying to quit smoking.

#### Feasibility

CONEMO is a nurse-supported app that utilizes behavioral activation to reduce depressive symptoms in patients with diabetes, hypertension, or both (39). The study by Lena Brandt et al. combines two feasibility pilot studies conducted in Lima, Peru to test the feasibility of: (1) implementing the app in two healthcare systems in Peru; (2) scaling up the app-based intervention. The study had 29 participants (mean age 60) utilize the app for 6 weeks, receiving three sessions per week. Semi-structured interviews revealed the app provided several health benefits, including reduction of stress and increase in motivation, and the majority were satisfied with the app. One major barrier was usability, with at least 72% of participants reporting some difficulties in using the app, though these subsided with longer use, and self-reported adherence was 50%. Participants also suggested the addition of audio. The study employed six nurses to support patients with app use and semi-structured interviews revealed that although they felt this was an innovative and helpful intervention, integration of CONEMO and daily responsibilities was challenging. Overall, CONEMO was found to be a feasible intervention.

CONEMO researchers also performed a composite study comprised of two pilot studies in Lima, Peru and one pilot study in São Paulo, Brazil with the dual goal of exploring the effectiveness of the CONEMO app and the feasibility to conduct a large randomized-control trial (39). The study enrolled 66 participants across the three sites. Data was collected actively: patients filled out a baseline PHQ-9, were then given access to CONEMO for 6 weeks, and filled a post-intervention PHQ-9, as well as passively: CONEMO system collected information such as sessions accessed and missed and interval between session access. Results of this study showed a general decrease in depressive symptoms based on decreasing PHQ-9 scores in 65–87% of participants, depending on the site, and a reduction in levels of functional disability. Challenges of this interventional study included difficulty with recruitment given that many patients were unable to read or write and a decline in session access over the course of the study.

Pratap et al. conducted a 3 month study to assess the feasibility of conducting a fully remote randomized controlled trial to screen for, assess, and treat depression in Latino individuals utilizing one of three apps to improve depressive symptoms: (1) EVO which uses therapeutic games, (2) iPST which employs psychotherapy principles, and (3) HTips which suggests mindfulness and behavioral exercises (40). The three apps were translated from English to Spanish by native Spanish speakers and professionals at Babble-on. The study enrolled 1,180 participants, but only 359 participated. Overall, feasibility of using mobile apps to remotely assess and treat depression was confirmed although the major challenges included a quick decline in app engagement and the higher cost and effort necessary to recruit Hispanic participants compared to non-Hispanic participants.

#### Intervention

Based on their feasibility findings, Pratap et al. then conducted a 3 month remote interventional study to compare recruitment and engagement of Hispanic and non-Hispanic participants and to compare treatment outcomes when participants utilized one of three apps: EVO, iPST, and HTips (42). The study remotely recruited and enrolled 1,020 participants, 389 of whom were Latino and 637 of whom were non-Latino. Participants were randomized to use one of the three apps for 3 months and PHQ-9 scores were collected at baseline and every week for the duration of the study. The study showed that PHQ-9 scores and self-reported disability scores decreased throughout the study without differences in recovery between Hispanics and non-Hispanics or by app used. The major challenges continued to be engagement, with Hispanic participants stopping the study nearly 2 weeks earlier than their counterparts and the high cost, high effort of enrolling Hispanic participants.

In this second interventional study (44), participants were recruited locally and nationally and randomized to one of three conditions for 8 weeks to address depressive symptoms: (1) ¡Aptívate! an app that uses behavioral activation, (2) iCouch which uses cognitive behavioral principles, and (3) treatment as usual, no app. The goals were to understand feasibility and efficacy of the ¡Aptívate! app. A total of 42 participants were enrolled (*n* = 22 ¡Aptívate!, *n* = 9 iCouch, *n* = 11 no app) and they self-reported app usage and completed the Spanish language Beck Depression Inventory-II (BDI-II) weekly. ¡Aptívate! is a self-help-based app and iCouch offers CBT. Results demonstrated lower depressive symptoms over time in those using the ¡Aptívate! app compared to treatment as usual, but no significant differences were found between ¡Aptívate! users and iCouch or between iCouch and treatment as usual. Challenges in this study included difficulties with local recruitment in South Carolina, although app engagement was higher in this group compared to nationally recruited participants.

In the third interventional study, researchers aimed to examine the mental health outcomes for Latinx Spanish-speaking patients with alcohol and other drug use (AOD) disorders with use of the CASA-CHESS mobile app for 8 weeks (43). This app was designed by translating and culturally adapting the theory-informed A-CHESS app into Spanish. The study enrolled 79 participants who were post-residential treatment for AOD, given a phone with the app, and followed for 6 months. Results show AOD symptoms for those that used the app for 4 months or longer were more stable with less use of illicit drugs, lower depressive and anxiety symptoms at 6 months compared to those who used the app for <4 months. Overall, this interventional study showed that an app can be an effective tool to provide continuity care for Spanish-speaking Latinos post-residential treatment. A major limitation of this work is the lack of app control or comparison group.

## Discussion

This review of the commercial market shows that there are many mental health apps available, but only a limited amount (14.5%) are offered in Spanish and none have conducted effectiveness studies with Spanish-speaking individuals. This problem is not unique to mental health apps. A study on apps for diabetes shows promise as it found that 30% (28/92) on the Android and Apple stores were in Spanish. However, when researchers investigated the Spanish readability of these apps, they found it was well above recommended reading levels, essentially making them inaccessible to many end-users (45). Although apps in Spanish may be growing in number, app developers must ensure that future apps are customizable, usable, and effective for the end-user populations.

Of the 10 apps studied in this literature review, only 4 apps (EVO, iPST, HTips, ¡Aptívate!) were translated (33, 42). This is likely as the others were developed in collaboration with Spanish speakers from their inception. However, Spanish-speakers in the United States are of course themselves diverse and thus cultural adaptation of apps must also consider: (1) end-user characteristics including nationality, locality, dialect, literacy level, socioeconomic status, (2) end-users' understanding of targeted conditions and associated stigmas, and (3) environments in which apps will be used and customizable features responsive to those environments. Of course, in many countries Spanish speakers are the majority and unique considerations for each culture, region, and clinical need must be considered.

User-centered design can help address most aspects of translation and cultural adaptation during the development phase, but it also requires intensive efforts and heavy upfront investment in recruitment, data collection, and usability testing. Another technique to achieve these goals is the utilization of cultural brokers to culturally adapt apps, as in the case of “Visit Planner,” an app aimed at helping Spanish-speaking patients prepare for their primary care appointments (46). However, this methodology could still prove challenging in some communities where cultural brokers may are not easily identifiable. Community collaboration and trust may be necessary preconditions to access and work with these individuals. Overall, the literature shows that translation is necessary, but not sufficient to guarantee the usability and effectiveness of an app. Developers and researchers should aim to use user-centered development techniques when possible, but more work on viable alternatives that require less investment for app translation and cultural adaptation are needed.

Feasibility studies have demonstrated that it is possible to implement mental health apps to treat depressive symptoms in Spanish-speaking patients in different healthcare systems and that it is possible to conduct studies that enroll large numbers of Spanish-speaking patients, even when done remotely. However, we interpret these studies show there are three major barriers researchers have to contend with: (1) quick decline in app engagement, (2) app usability for some segments of the population, and (3) Increased expense and labor required to recruit Spanish-speaking participants.

User-centered design has been utilized as a method to empower potential end-users to contribute to app development and thus try to counteract usability and engagement concerns (25). The three feasibility studies discussed in this review did not describe the use of a user-centered design which may have contributed to a quick decline in app engagement. However, the studies also show that to counter engagement issues, app use should be paired with primary care, be accompanied by supportive nursing, or include features that help patients connect to mental health services. Nursing or other support staff, such a promotoras, can also help address usability issues in populations who may need extra technical help. Recruitment challenges may be multifaceted and may therefore require dynamic solutions. Work by Stuart Winter et al. describe best practices for including minorities in research including improving incentives, partnering with key community organizations, understanding the most used and effective methods of communication, addressing issues of distrust, and adding cultural brokers to the research team (26). Further work is needed to assess optimal incentives for Spanish-speaking populations and identify barriers to engagement in remote mental health studies.

Early data from interventional studies identified in this review demonstrate that mental health apps in Spanish may be effective tools to assess and treat depressive symptoms and help patients remain stable post-residential treatment for alcohol and AOD. Though the published literature on intervention studies is nascent, they show promising evidence that mobile health interventions can be successful for Spanish-speaking patients. This is particularly important given that these interventions will improve access to care for many patients in their native language, address the stigma associated with mental health conditions, and ultimately help close the gap on mental health disparities for Spanish-speaking patients. However, in the past 5 years few studies in the literature have appeared that more work needs to be done to increase research in this area. More apps need to be translated, culturally adapted and tested for feasibility and usability. Future efforts for the 32 apps available in Spanish identified in the market review should involve testing their effectiveness in this population. From these investigations, simple guidelines could be created to help patients, primary care providers, and/or mental health providers select the most appropriate app for each individual based on their characteristics, health literacy level, environment of use, personal preferences, and mental health condition.

Although the number of mental health apps available in Spanish is growing, translation of these apps alone may not be enough to improve access to these tools. A recent study of Medicare patients suggests that 38% of Spanish speakers do not have access to a smartphone with a data plan for wireless internet, precluding app use for many (27). A less visible but prevalent barrier to access is the lack of comfort with technology. The digital divide related to access to smartphones has also evolved into a lack of digital skills to navigate technology (28). Thus, Spanish-language apps are only as accessible as the users' digital literacy and comfort. While beyond the scope of this paper, we have seen this in our clinical work and as we have work to create a Spanish version of our teams' own open source mental health app (47, 48). To combat this, we have developed Digital Opportunities for Outcomes in Recovery Services (DOORS) to help people gain the core competencies and functional skills necessary to utilize digital health tools effectively (49). While DOORS is currently available in English, efforts to create partners and a team to build a Spanish version are underway. Future Spanish-language app development should consider the literacy levels of users and ponder what level of technical competency is necessary to utilize the tool.

As with any study, this review has several weaknesses that must be addressed. First, the market search was conducted on a database with 220 apps which is the largest database of mental health apps, however app stores host thousands of mental health-related apps. Thus, the percentage of apps available in Spanish may not be indicative of the entire landscape. Second, no single search term can discover all applicable articles on this topic. These results may also be influenced by publication bias due to apps or studies that may exist but have yet to be published. Third, while this was a U.S. based study in terms of the app marketplaces reviews and thus results may not generalizable to other countries and Spanish speakers, we note we did review research from around the world. Fourth, we looked at papers from the last 5 years which covers the majority of app research but will have missed earlier works.

## Conclusion

Our study reveals that despite increasing interest in Spanish-language apps to address mental health, the commercial marketplace is not currently meeting the demand. None of the apps we reviewed on the marketplace were found in the academic literature, reflecting a gap between research and commercially available apps today. User-centered development design emerged as a leading strategy to increase the number of apps that are linguistically and culturally adapted to the Spanish-speaking population. Interventional studies show promising evidence that apps can help address mental health condition if challenges surrounding engagement and app usability are overcome. This study highlights that through thoughtful design and development, apps may hold the key to reducing mental health disparities in Spanish-speaking Hispanics in the United States.

## Author Contributions

JT and AM conceptualized the study. EC and AM reviewed the literature. AM led the paper drafting. All authors discussed paper selection, edited multiple drafts, and reviewed the final paper.

## Conflict of Interest

JT reports unrelated research support from Otsuka. The remaining authors declare that the research was conducted in the absence of any commercial or financial relationships that could be construed as a potential conflict of interest.

## Abbreviations

*m*Health: mobile health care
apps: mobile applications
AOD: alcohol and other drug use.
